# Treatment of thoracolumbar burst fractures by short-segment pedicle screw fixation using a combination of two additional pedicle screws and vertebroplasty at the level of the fracture: a finite element analysis

**DOI:** 10.1186/s12891-017-1623-0

**Published:** 2017-06-15

**Authors:** Jen-Chung Liao, Weng-Pin Chen, Hao Wang

**Affiliations:** 1 Department of Orthopedic Surgery, Bone and Joint Research Center, Chang Gung Memorial Hospital, Chang Gung University, Taoyuan, Taiwan; 2 Department of Mechanical Engineering, National Taipei University of Technology, 1, Sec. 3, Chung-Hsiao E. Rd, Taipei, 10608 Taiwan

**Keywords:** Thoracolumbar burst fracture, Posterior instrumentation, Vertebroplasty, Finite element analysis

## Abstract

**Background:**

Traditional one-above and one-below four-screw posterior short-segment instrumentation is used for unstable thoracolumbar burst fractures. However, this method has a high rate of implant failure and early loss of reduction. The purpose of this study was to use finite element (FE) analysis to determine the effect of treating thoracolumbar burst fractures by short-segment pedicle screw fixation using a combination of two additional pedicle screws and vertebroplasty at the level of the fracture.

**Methods:**

An intact T11-L1 spine FE model was created from the computed tomography images of a male subject. Four fixation models with posterior fusion devices (pedicle screws, rods, cross-link) were established to simulate an unstable thoracolumbar fracture with different fusion surgeries: short-segment fixation with: 1) a link (S-L); 2) intermediate bilateral screws (S-I); 3) a link and calcium sulfate cement (S-L-C); 4) intermediate bilateral screws and calcium sulfate cement (S-I-C). Different loading conditions (flexion, extension, lateral bending, and axial rotation) were applied on the models and analyzed with a FE package. The range of motion (ROM), and the maximum value and distribution of the implant stress, and the stress in the facet joint, were compared between the intact and fixation models.

**Results:**

The ROM in flexion, extension, axial rotation, and lateral bending was the smallest in the S-I-C model, followed by the S-I, S-L-C, and S-L models. Maximum von Mises stress values were larger under lateral bending and axial rotation loadings than under flexion and extension loading. High stress was concentrated at the crosslink and rod junctions. Maximal von Mises stress on the superior vertebral body for all loading conditions was larger than that on the inferior vertebral body. The maximal von Mises stress of the pedicle screws during all states of motion were 265.3 MPa in S-L fixation, 192.9 MPa in S-I fixation, 258.4 MPa in S-L-C fixation, and 162.3 MPa in S-I-C fixation.

**Conclusions:**

Short-segment fixation with two intermediate pedicle screws together with calcium sulfate cement at the fractured vertebrae may provide a stiffer construct and less von Mises stress of the pedicle screws and rods as compared to other types of short-segment fixation.

## Background

Burst fractures account for approximately 20% of thoracolumbar fractures, and occur due to an axial loading force that results in failure to support the anterior and middle column [1, 2]. Surgery is usually indicated when there is a severe deformity, and/or neurologic deficit. Whether anterior or posterior surgery is the most effective treatment for burst fractures remains controversial. Some authors advise anterior surgery to remove retropulsed fragments [3, 4], but posterior surgery is popular because it is an easier approach and allows clearance of the spinal canal by ligamentotaxis.

One-above and one-below posterior short-segment instrumentation with fusion has been widely used for unstable thoracolumbar burst fractures for the past 3 decades [5]. Pedicular instrumentation enables kyphotic correction, indirect reduction of canal encroachment, and early mobilization. However, this method has a high rate of implant failure, and early loss of reduction because of loss of anterior support [6]. Over the past decade, some studies have demonstrated that augmentation of the fractured vertebra with absorbable bone cement could enhance fracture union and prevent implant failure. Liao et al. [7] and Korovessis et al. [8] demonstrated that injectable calcium sulfate cement or injectable calcium phosphate cement used as a transpedicular grafting material in thoracolumbar fractures could obtain clinical and radiographic results equal to autogenous cancellous bone graft.

In recent years, biomechanical and clinical studies have suggested that two additional screws inside the fractured vertebra could improve stability and provide better kyphotic correction. In a biomechanical study, Norton et al. [9] showed that two additional screws in the fractured vertebra (a six-screw construct) of an unstable thoracolumbar burst fracture could increase the stiffness of the implant, and reduce stress on each pedicle screw, as compared to a four-screw construct. However, there have been no studies examining the effect of augmentation by a combination of screws and bone cement at the fractured vertebra, and comparing the effect of this fixation method with other types of posterior short-segment instrumentation for thoracolumbar burst fractures.

We hypothesize that posterior short-segment instrumentation with fractured vertebra augmentation by a combination of vertebroplasty and two additional screws can provide a stronger construct than other types of posterior short-segment instrumentation for thoracolumbar burst fractures. In the current study, we established a finite element (FE) model of thoracolumbar burst fractures, and four posterior short-segment fixation methods were tested. The purpose was to determine the most optimal methods for the treatment of thoracolumbar burst fractures.

## Methods

### Models

**Fig. 1:**
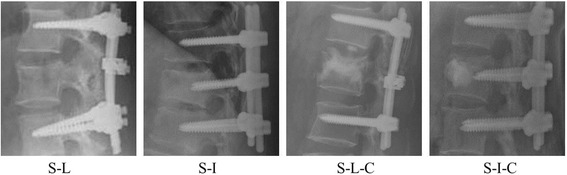
Four types of fixation. S-L: four screws with a link. S-I: six-screw construct. S-L-C: four screws with a link, and calcium sulfate bone cement vertebroplasty. S-I-C: six screws with calcium sulfate bone cement vertebroplasty

Four fixation models with posterior fusion devices (pedicle screws, rods, cross-link) were established to simulate an unstable thoracolumbar fracture with different fusion surgeries: short-segment fixation with: 1) a link (S-L); 2) intermediate bilateral screws (S-I); 3) a link and calcium sulfate cement (S-L-C); 4) intermediate bilateral screws and calcium sulfate cement (S-I-C). Radiographs representing these four types of fixation are shown in Fig. 1.

### Establishment of FE model of an intact normal thoracolumbar spine

**Fig. 2:**
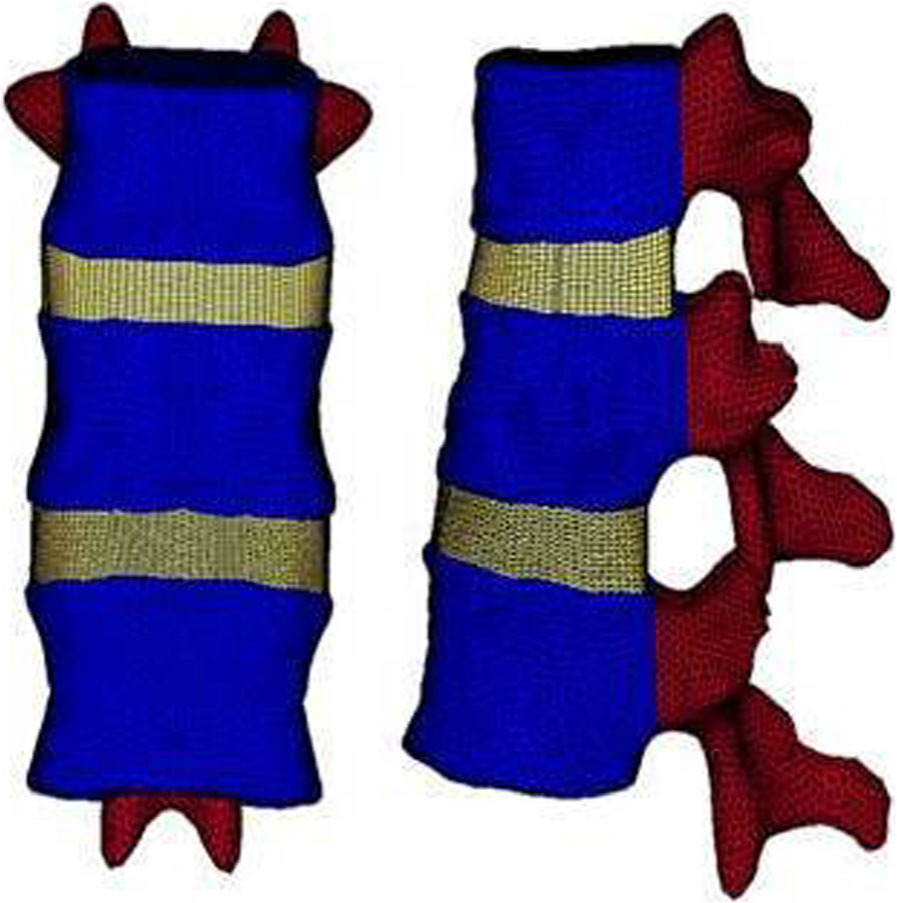
T11-L1 finite element model

A FE model of an intact normal spine was developed based on 1-mm-interval cross-sectional computed tomography (CT) spine images of a 55-year-old male provided by the US National Library of Medicine (NLM; NIH, Bethesda, MD). The FE model of the thoracolumbar section from T10 to L2 was established as follows. 1) A series of CT scans were imported into Amira 4.1 software (Visage Imaging, Carlsbad, CA). The vertebral boundaries as regions of interest (ROI) were identified on multiple images to form a three-node triangular surface model. 2) The surface model was imported into SolidWorks (SOLIDWORKS Corporation, Boston, MA), to further reconstruct a three-dimensional solid model of the thoracolumbar spine. 3) The solid model of the thoracolumbar spine was imported into HyperWorks 10.0 (Altair Engineering, Inc., Troy, MI) to form an eight-node hexahedral FE model. Since CT scanning does not provide the soft tissue contours of the intervertebral discs, the geometric characteristics of intervertebral discs were created as previously described [10]. The volume ratio of the annulus fibrosus to the nucleus pulposus was set to 6:4, the thickness of the cortical bone was set to 1 mm, and the endplate was set to 0.5 mm [10]. 4) The final FE model was imported into Abaqus FE analysis software (Abaqus/CAE v.6.10; Simulia Corp., Providence, RI) for analysis (Fig. 2).

### Establishment of FE models of injured vertebra

**Fig. 3:**
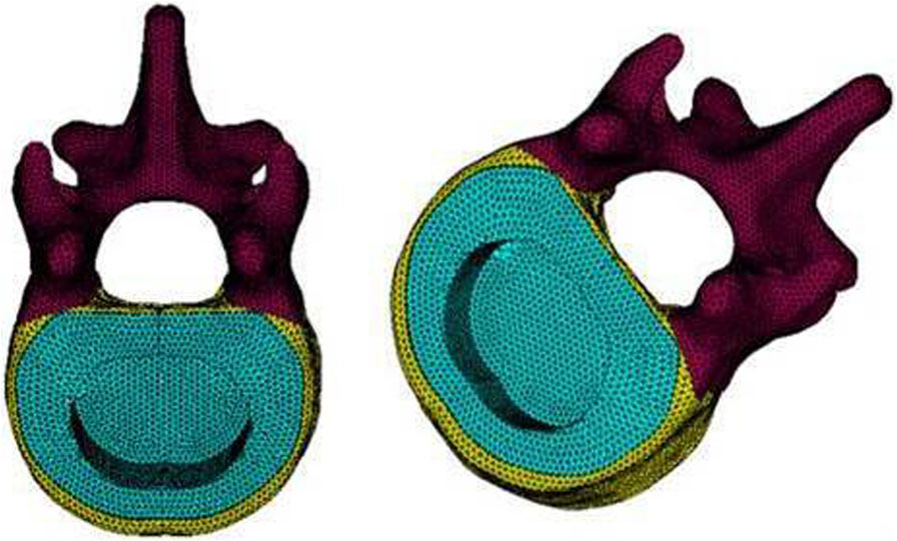
T12 injured vertebrae finite element model

In this study, the vertebral resection (corpectomy) method was used to simulate a vertebra fracture. Briefly, one-half of the sponge bone of the T12 vertebra was removed to weaken the vertebral strength; the structure of the posterior portion was reserved to establish a FE model of an unstable thoracolumbar fracture (Fig. 3).

### Establishment of solid internal fixator models

#### Pedicle screw system

**Fig. 4:**
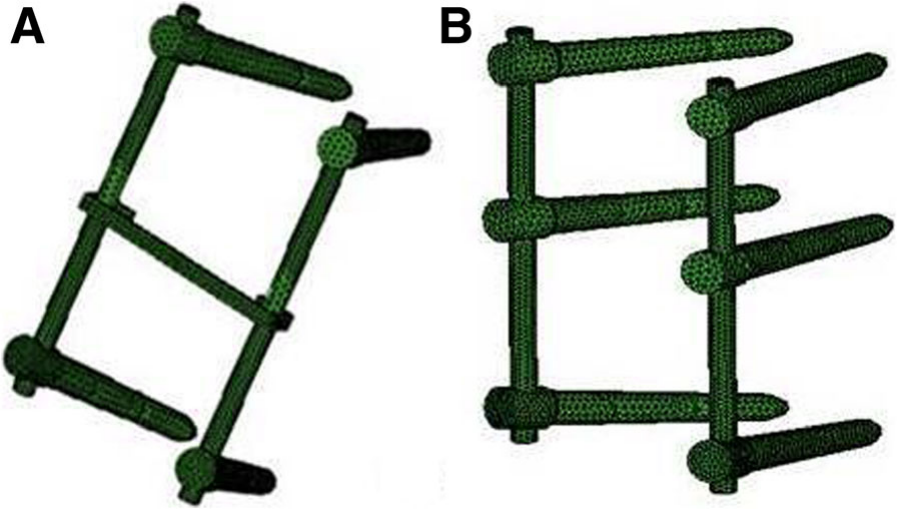
Finite element models of internal fixations. **a**: Four screws with a link. **b**: Six-screw construct

The pedicle screw system in this study was modeled based on the SmartLoc Spinal Fixation System (A-SPINE Asia Co., Ltd.). Using physical measurements and 3D reverse engineering, a solid model was created using Solidworks. The length of the screw was 45 mm, the radius was 2.4 mm, and the cross-sectional area was 18.1 mm^2^. The length of the vertical connecting rod was 80 mm, the radius was 2 mm, and the cross-sectional area was 12.6 mm^2^. The horizontal connecting rod was 35 mm long, 7 mm wide, and 5 mm high. In order to shorten the FE analysis time, the screw thread part and the vertical connecting rod middle connection part of the structure were neglected. After the solid models of the implants were created, they were imported into HyperWorks to generate the FE meshes. The four-vertebral pedicle screw plus transverse connecting rod FE mesh model is shown in Fig. 4a, and the six-vertebral pedicle screw model is shown in Fig. 4b.

#### Four fixation models

**Fig. 5:**
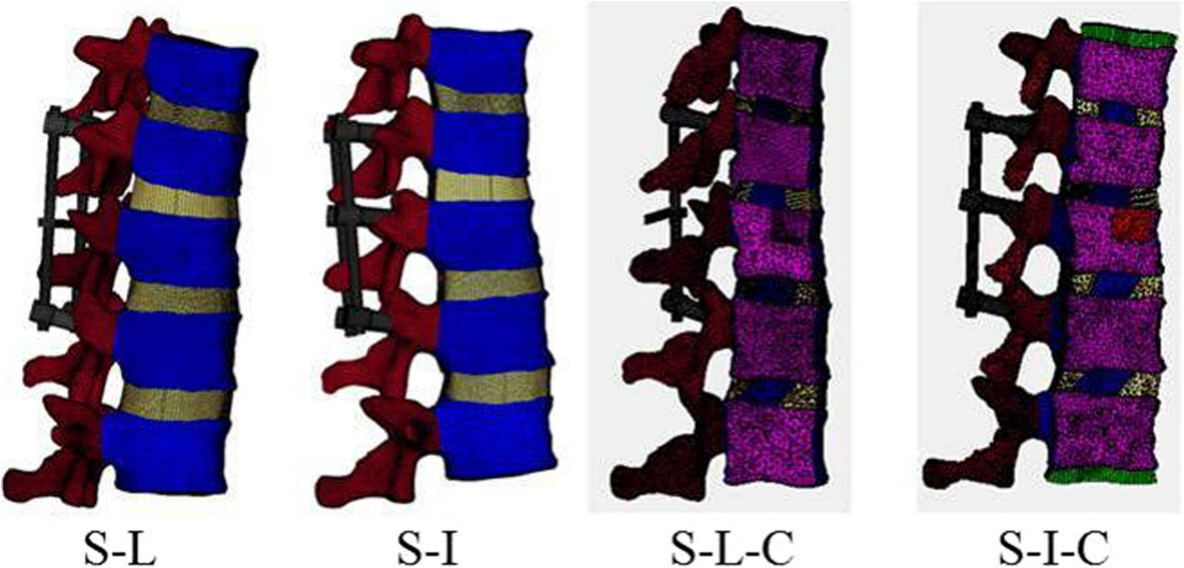
Four-screw fixation finite element model with T11-L1 spine segments. S-L, short-segment pedicle screw fixation with a link; S-I, short-segment pedicle screw fixation with two intermediate screws; S-C, short-segment pedicle screw fixation with cement vertebroplasty; S-I-C, short-segment pedicle screw fixation with two intermediate screws plus cement vertebroplasty

The internal fixators were placed in the thoracolumbar models as described by Park et al. [11]. Four fixation models were used for comparison in this study: S-L, S-I, S-L-C, S-I-C. FE models of these four fixation types are shown in Fig. 5.

#### Material parameter settings

The material parameters (Young’s moduli and the Poisson’s ratios of cortical bone, cancellous bone, posterior bone element, endplate, and intervertebral disc) of the thoracolumbar models were set based on the work of Qiu et al. [12]. The models were assumed to be homogeneous, isotropic, and linearly elastic. Material parameters are summarized in Table 1.

**Table 1:** The material properties of the finite element model

Component name	Young’s modulus (Mpa)	Poisson ratio, y	Element type
Cortical bone	12,000	0.3	tetrahedral
Cancellous bone (normal)	345	0.2	tetrahedral
Cancellous bone (osteoporosis)	70	0.2	tetrahedral
Cement (Calcium sulfate)	147	0.3	tetrahedral
Posterior element	3500	0.25	tetrahedral
Endplate	12,000	0.3	tetrahedral
Disc-annulus	4.2	0.45	Prism
Disc-nucleus	6	0.499	Prism
Annuls fiber	500	0.3	tri
Rigid body	10,000,000	0.4	tetrahedral
Titanium	110,000	0.3	tetrahedral

#### Loading and boundary conditions, and friction coefficient settings

The loading and boundary conditions of the FE analysis were based on the work of Qiu et al. [13]. Flexion, extension, lateral bending, and axial rotation were applied to the rigid plane above the T11 vertebra, respectively, and the load was applied as a set in a progressive manner (2.5 Nm, 5.0 Nm, 7.5 Nm of bending moment), respectively, and all degrees of freedom on the bottom nodes of L1 were constrained. To simulate a real contact setting, the friction coefficient of the contact surface of the facet joint was set to 0.1. This study simulated bone fusion following surgery, therefore, the interfaces between the screw and vertebrae were set to be perfectly bonded.

#### Assessment indexes

By evaluating the range of motion (ROM) of T11-L1, the maximum von Mises stress on the pedicle screw root, and stress distribution of the pedicle screws and rods of the four fixation FE models under flexion, extension, left/right lateral bending, and left/right axial rotation, the biomechanical differences between the four fixation models could be compared.

## Results

### Validation of the intact T11-L1 FE model

After establishment of the normal T11-L1 FE model, moments of 7.5 Nm in six directions were obtained. The ROM of the T11-T12 segment were: flexion (3.0°), extension (3.1°), left bending (3.3°), right bending (3.8°), left rotation (2.2°), and right rotation (2.0°). The ROM of the T12-L1 segment were: flexion (3.3°), extension (3.7°), left bending (4.0°), right bending (3.6°), left rotation (1.3°), and right rotation (1.4°) (Table 2). The ROM results were similar to those reported by Panjabi et al. [14].

**Table 2:** Comparison between the present intact model and that of Panjabi et al. [12]

Loading mode	T11-T12		T12-L1	
	Panjabi study	Present study	Panjabi study	Present study
Flexion (7.5 Nm)	2.7° ± 1.3°	3.0°	2.9° ± 1.4°	3.3°
Extension (7.5 Nm)	2.4° ± 1.3°	3.1°	3.9° ± 1.4°	3.7°
Left bending (7.5 Nm)	3.5° ± 1.1°	3.3°	3.7° ± 1.1°	4.0°
Right bending (7.5 Nm)	3.5° ± 1.1°	3.8°	3.7° ± 1.1°	3.6°
Left rotation (7.5 Nm)	1.8° ± 0.7°	2.2°	1.2° ± 0.7°	1.3°
Right rotation (7.5 Nm)	1.8° ± 0.7°	2.0°	1.2° ± 0.7°	1.4°

### ROM of the four FE fixation models

The moments of 7.5 Nm in six directions were applied to the four fixation models. The ROM of the T11-L1 segment were: S-L group: flexion (1.03°), extension (1.05°), left bending (2.92°), right bending (2.80°), left rotation (2.60°), right rotation (2.60°); S-I group: flexion (0.95°), extension (0.98°), left bending (2.67°), right bending (2.54°), left rotation (2.32°), right rotation (2.32°); S-L-C group: flexion (0.98°), extension (1.00°), left bending (2.75°), right bending (2.61°), left rotation (2.46°), right rotation (2.46°); S-I-C group: flexion (0.87°), extension (0.92°), left bending (2.57°), right bending (2.51°), left rotation (2.23°), right rotation (2.23°) (Table 3).

**Table 3:** Range of motion of four fixations with different loading

Loading mode	S-L	S-I	S-L-C	S-I-C
Flexion	1.03°	0.95°	0.98°	0.87°
Extension	1.05°	0.98°	1.00°	0.92°
Left bending	2.92°	2.67°	2.75°	2.57°
Right bending	2.80°	2.54°	2.61°	2.51°
Left rotation	2.60°	2.32°	2.46°	2.23°
Right rotation	2.60°	2.32°	2.46°	2.23°

### Comparisons between the four fixation models and the intact model

**Fig. 6:**
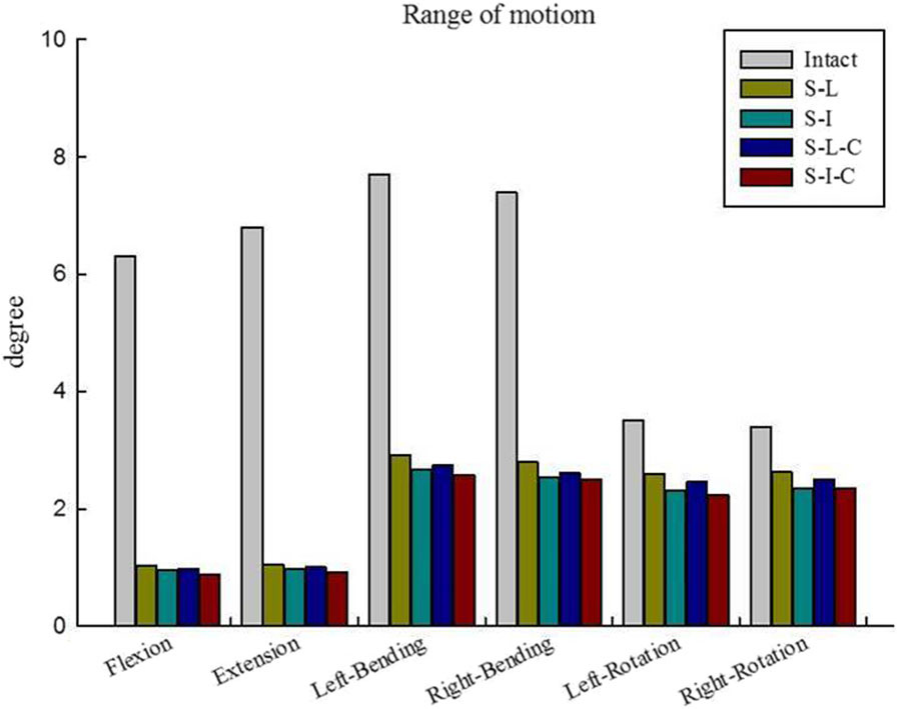
Range of motion (ROM). Compared to the intact normal spine model, the four fixation models showed a decreased ROM in all directions. S-I-C fixation had the lowest ROM

The percentage of ROM of the four fixation models with respect to the intact model was compared in terms of the degree of ROM in each direction of movement. The percentage of ROM of the S-I-C group was lowest in all directions of motion (flexion: 13.2%; extension: 13.5%; left bending: 35.0%; right bending: 33.9%; left rotation: 64.6%; right rotation: 65.6%), indicating the stability was the best. The percentage ROM of the S-L group was the highest in all directions of motion (flexion: 15.6%; extension: 15.4%; left bending: 39.7%; right bending: 37.8%; left rotation: 75.3%; right rotation: 76.5%), indicating the worst stability. The S-I group ranked second, and the S-L-C group ranked third with respect to stability (Fig. 6).

### Stress distribution of the pedicle instrumentation system

**Fig. 7:**
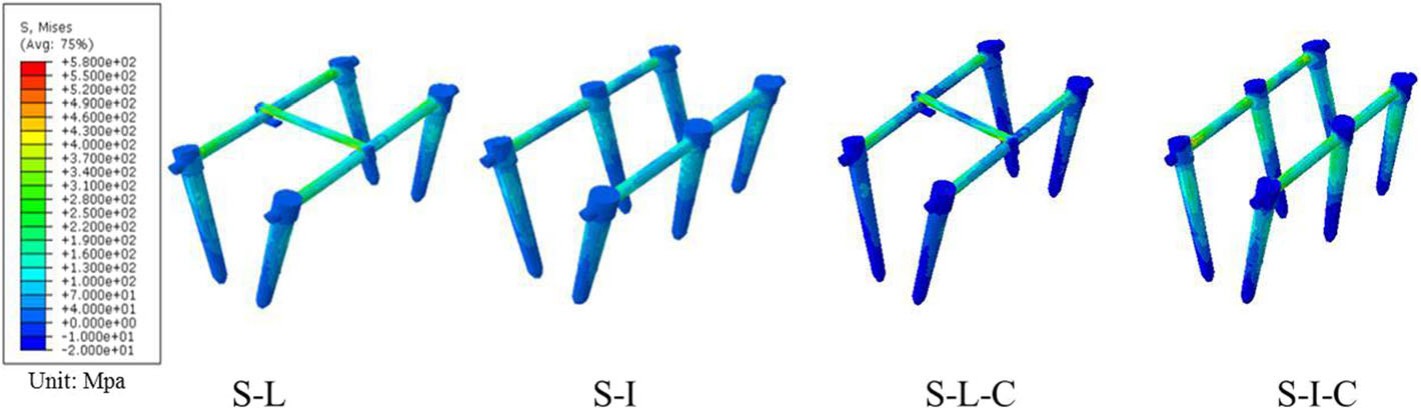
Stress nephogram of the pedicle screws and rods with rotation stress

von Mises stress distribution analysis showed that high stresses were mainly concentrated on the screws during flexion and extension. High stress in lateral bending was mainly concentrated on the longitudinal connecting rod. High stress was also concentrated at the junction of the transverse connecting rod and the longitudinal connecting rod under a rotation load (Fig. 7).

### Maximum von Mises stress on the root of the pedicle screw

von Mises stresses of the pedicle screw neck of the S-L group were 30.9 MPa in flexion, 31.7 MPa in extension, 200.7 MPa in left lateral bending, 202.3 MPa in right lateral bending, 261.9 MPa in left rotation, and 265.3 MPa in right rotation. Stresses in the S-I group were 24.7 MPa in flexion, 25.7 MPa in extension, 141.8 MPa in left lateral bending, 144.3 MPa in right bending, 192.9 MPa in left rotation, and 191.2 MPa in right rotation. Stresses in the S-L-C group were 25.9 MPa in flexion, 26.1 MPa in extension, 156.5 MPa in left lateral bending, 258.4 MPa in right lateral bending, 200.4 MPa in left rotation, and 201.6 in right rotation. Stresses in the S-I-C group were 23.5 MPa in flexion, 24.6 MPa in extension, 128.6 MPa in left lateral bending, 130.2 MPa in right lateral bending, 162.3 MPa in left rotation, and 162.3 MPa in right rotation (Table 4).

**Table 4:** Maximum von Mises stress on the root of the pedicle screw

Loading mode	S-L	S-I	S-L-C	S-I-C
Flexion	30.9 MPa	24.7 MPa	25.9 MPa	23.5 MPa
Extension	31.7 MPa	25.7 MPa	26.1 MPa	24.6 MPa
Left bending	200.7 MPa	141.8 MPa	156.5 MPa	128.6 MPa
Right bending	202.3 MPa	144.3 MPa	258.4 MPa	103.2 MPa
Left rotation	261.9 MPa	192.9 MPa	200.4 MPa	162.3 MPa
Right rotation	265.3 MPa	191.2 MPa	201.6 MPa	162.3 MPa

## Discussion

Surgical management of a thoracolumbar burst fracture varies according to many factors. Fracture morphology, neurologic status, and surgeon preference all play major roles in deciding the surgical approach. An anterior approach to the fracture site can directly decompress the neural elements, and the reconstruction ca be performed by simultaneous iliac bone graft and plating. Hitchon et al. [15] and Sasso et al. [16] reported that the anterior approach was superior to the posterior approach in its ability to maintain the kyphotic correction at final follow-up, but both approaches achieved similar in clinical results. A major disadvantage of anterior surgery is potential donor site morbidity from harvesting iliac tri-cortical bone graft. In addition, the cost of the anterior approach is generally greater than that of the posterior approach. We usually use a posterior approach for the repair of thoracolumbar burst fractures, which is one of the reasons that led to the current study.

Traditional one-above and one-below short-segment posterior instrumentation has the potential for early implant failure and re-kyphosis due to lack of anterior support of the defect inside the injured vertebra. Several techniques, such as reinforcement with additional screws at the fractured level, or augmentation with any kind of bone cement to fill the defect of the fractured vertebra, have been proposed to improve the stability of the posterior instrumentation construct, and thus prevent implant failure and enhance fracture union [17–20]. However, no studies examined reinforcement of a fractured vertebra with a combination of bone cement with two additional fracture-level screws. Several biomechanical studies have suggested that reinforcement with fracture-level screws could improve the biomechanical stability of the construct [21–23]. Clinical studies have also suggested that reinforcement with additional screws at the fractured level can provide better kyphotic correction, more effectively restore fractured vertebra height, and allow earlier ambulation for patients with thoracolumbar burst fractures [24, 25]. On the contrary, authors have also claimed that a fractured vertebra augmented by absorbable bone cement followed by a posterior short-segment construct can provide satisfactory clinical results, with a low implant failure rate of 0% to 5% [19, 20]. Xu et al. [26] designed a FE model of a thoracolumbar burst fracture, and demonstrated that vertebroplasty inside the fractured vertebra can significantly reduce the stresses of the pedicle instrumentations and spine to prevent kyphotic correction loss and implant failure. In a clinical study, Liao et al. [27] showed that two screws placed inside the fractured vertebra with short-segment instrumentation was associated with shorter surgical time and less implant failure as compared to augmentation with injectable calcium sulphate/phosphate cement following posterior short-segment instrumentation.

In the current study, the four fixation models all demonstrated decreased ROM in all directions as compared with the intact spine model. ROM in flexion, extension, axial rotation, and lateral bending was the smallest in the S-I-C fixation model, followed by the S-I and S-L-C fixation models; the greatest ROM was seen in the S-L fixation model. The S-I-C and the S-I fixation model both contained six screws in the construct; the S-L-C and the S-L fixation only had four screws in the construct. Reinforcement with two additional screws at the fractured level can enhance biomechanical stability; and these results were similar to a previous FE study by Li et al. [28]. Two additional screws inside the fractured vertebra was also stronger than when bone cement was used inside the fractured level (S-I fixation model versus S-L-C fixation model). This result was similar to the clinical results demonstrated by Liao et al. [27].

The pedicle screw is considered to be the weakest point of the posterior fixation, and implant failure is usually caused by screw loosening or screw root breakage [29]. It has also been shown that the pedicle screws still continue to bear most of the load after fusion [30, 31]. To prevent early screw breakage, and to prolong the life of the screw, it is necessary to prevent the load on the screw from reaching the fatigue load. For this reason, we evaluated the maximum von Mises stress on the root of the pedicle screws in the four fixation models. A lower stress distribution around the root of the pedicle screw means the less chance of pedicle screw breakage. The maximum von Mises stress of the pedicle screws in the S-L group was higher than that in the other three fixation groups. In the S-I-C group, the maximum von Mises stress of the pedicle screw in all directions was the lowest. These results indicate that using two additional screws inside the fractured vertebra can reduce the stress on the pedicle screw root. Further augmentation with bone cement at the fractured level can further reduce the stress around the pedicle screw root, and further decrease the probability of screw failure. In addition, we found the pedicle screw root under lateral bending and axial rotation load sustained higher von Mises stresses, as finding similar to that of Xu et al. [32].

Injectable bone substitutes such as calcium sulfate, calcium phosphate cement, and hydroxyapatite cement are widely used for filling bone defects. These bone substitutes not only have osteoconductive ability to promote bone union, but also provide initial mechanical support. Evaniew et al. [33] used calcium sulfate/phosphate cement to manage patients with bone defects after curettage of primary tumor. Orsini et al. [34] demonstrated that calcium sulfate cement could promote new bone formation in a rabbit model of bone defects. Furthermore, Xu et al. [26] used a FE model to show that cement augmentation of a fractured vertebra could decrease the von Mises stress on the rods by 50% and on the screws by 40%. In the current study, however, the initial stability provided by bone cement inside the fractured vertebra did not achieve the stability provided by two additional screws. Nevertheless, an advantage of bone cements is that they can stimulate bone healing inside the vertebra, which screws cannot provide. Therefore, a combination of bone cement and two additional screws inside the fractured vertebra along with short-segment instrumentation may be an ideal surgical method for thoracolumbar burst fractures because provides greater initial stability, and also stimulates bone growth.

There are limitations of this study that should be considered. First, the FE spine models were reconstructed from data of a single patient, and thus is not representative of various ages or different sexes. Second, most cases of thoracolumbar burst fractures are associated with injury to the upper endplate and/or adjacent intervertebral disc. This was not addressed in the current models. Removal of the upper end-plate of the vertebrae in the FE model may better reflect the real injury pattern of a burst fracture. However, the main purpose of this study was to compare different fixation techniques, not different injuries. Third, because the current FE study only provided comparison data for the stability of different fixation methods, and the material representations of the biological structures were assumed to be linearly elastic, biomechanical studies of cadavers are necessary to validate the results for clinical practice. If the results are validated in cadaver studies, clinical cohort studies would be warranted.

## Conclusion

Additional bilateral pedicle screws combined with cement augmentation vertebroplasty at the level of the fractured vertebra results in a stiffer construct and lower von Mises stress on the pedicle screws as compared with other types of posterior short-segment fixations. Four-screws plus a link resulted in the highest stress on the screws, suggesting this method would be associated with a higher incidence of implant failure and re-kyphosis. The S-I-C fixation was the strongest posterior short-segment fixation method for thoracolumbar burst fractures.

## Abbreviation

FE: Finite element
ROM: Range of motion
